# The most impactful findings on liver cancer in 2023

**DOI:** 10.1097/HC9.0000000000000398

**Published:** 2024-03-11

**Authors:** Jin Lee, Kaisa L. Hanley, Gen-Sheng Feng

**Affiliations:** 1 Department of Pathology, School of Medicine, University of California San Diego, La Jolla, California, USA; 2 Department of Molecular Biology, School of Biological Sciences, University of California San Diego, La Jolla, California, USA

HCC is associated with chronic liver diseases such as HBV and HCV infections, cirrhosis of various etiologies, metabolic dysfunction–associated fatty liver disease and metabolic dysfunction–associated steatohepatitis (formerly known as NAFLD and NASH). HCC is a leading cause of cancer death in patients, and its incidence has been rising in most countries.^1^ Currently, for early-stage liver cancer, curative therapies include surgery (resection/partial hepatectomy) and liver transplant. However, HCC is often found at late stages and systemic therapy for advanced cases is still limited, with no guidelines for patient stratification and substantial side effects and off-target issues that together lead to reduced survival rates. Therefore, improved therapy for late-stage HCC is urgently needed to improve the chance of survival.^2^

Sorafenib (Nexavar), a multityrosine kinase inhibitor, was introduced in 2007 as the first option of systemic therapy for advanced HCC. Although it was an effective systemic treatment for more than 10 years, the improved survival rate is only 3–6 months. Inspired by the success of immune checkpoint blockades (ICBs) in melanoma, ICBs have been extensively tested in numerous cancer types including HCC. In 2020, the Food and Drug Administration approved ICB as additional systemic therapies [Nivolumab and Pembrolizumab for programmed cell death protein 1 (PD-1) blockades; ipilimumab for cytotoxic T lymphocytes antigen-4 (CTLA-4)] for advanced HCC. Overexpression of PD-1 and CTLA-4 are hallmarks of the immunosuppressive environment displayed in liver cancer. Immunotherapy-based regimens show effective tumor killing and improve survival in patients. However, only 15% to 20% of patients respond to ICBs, and many initial responders show tumor relapse, perhaps due to a limited understanding of ICB’s tissue-specific mechanisms. Moreover, even within the responder cohort, patients have shown unexpected death or side effects by immune-mediated adverse events. Thus, the design of ICB treatments for patients with HCC need better strategies to improve survival rates while avoiding immune-mediated adverse events.

One reason for the failure of ICBs could be that we underdetermine the tissue-specific tumor microenvironment (TME) in response to immunotherapy. In general, immune cells develop in specialized lymphoid organs, but they are maintained and function in tissue sites throughout the body. The adult liver is usually considered a nonimmunological organ that primarily functions for metabolic, nutrient storage, and detoxification activities. However, more recent work highlights an appreciation of the liver as an immunological organ. The liver displays unique anatomical and physiological features, receiving both oxygen-rich blood from the hepatic artery and nutrient-rich blood from the portal vein.^3^ Liver-resident immune cells develop as the liver matures and populate liver sinusoids. These can help to maintain homeostasis in infectious disease and HCC by complex immunological activities mediated by a diverse repertoire of immune cells. Therefore, we must understand the tissue-specific liver immune microenvironment to design better ICB-based treatment strategies in patients with HCC.

Throughout the year 2023, a new era of spatial -omics, combining spatial transcriptomics and multiplex antibody staining against immune cells, has advanced the field of cancer immunology.^4^ Previously, immune research at the single-cell level used flow cytometry, and single-cell (sc)-RNA sequencing could denote immune cell populations with distinct gene expression profiles. However, it couldn’t show direct or indirect cell-to-cell spatial interactions in the liver-specific immune microenvironment. These new techniques allow us to see how immune cells move during cancer development and ICB treatment and reveal the physical interactions of different immune components. Thanks to developing techniques, preclinical studies can even identify novel subpopulations and elucidate the unique liver cancer microenvironment. Based on the current status of ICBs in the clinic and these technical advances in basic science, we considered that several impactful papers published in the past year are particularly exciting and represent substantial steps toward a better understanding of the liver tumor immune microenvironment (TIM).

Ruf et al^5^ performed 37-plex co-detection by indexing imaging with integrating comprehensive single-cell-RNA sequencing from immune cells in patients with HCC. Co-detection by indexing is a fluorescence microscopy platform based on the detection of DNA-conjugated antibodies. With beautiful spatial images to denote immune cells in TME, this paper unveiled one distinct cluster of mucosal-associated invariant T (MAIT) cells within the T cell compartment in human HCC samples. MAIT cells represent an abundant innate-like T cell subtype in the human liver. This study was the first to show a function of MAIT cells in human HCC, with MAIT cell infiltration into liver tumors consistently and significantly impaired. They showed high expression of PD-1 and T cell immunoglobulin and mucin domain-containing protein 3 (TIM-3) with lack of proinflammatory cytokine release and reduced frequency of granzyme B. This impairment of MAIT was attributed to interaction with tumor-associated macrophages expressingprogrammed death-ligand 1 (PD-L1) in adjacent liver cancer areas. Impairment of MAIT cell phenotypes was confirmed in response to tumor growth using several syngeneic models of murine orthotopic liver cancer. Anti-PD-L1 ICB restored MAIT cell cytotoxicity to reduce tumor growth. This finding suggested that MAIT cells will be a novel potential target for aPD-1/aPD-L1-based ICB in patients with liver cancer.

In another finding for under-represented immune cell populations in response to ICBs, Xue and colleagues investigated functions of neutrophil heterogeneity in tumor control. Neutrophils are a smaller population in mouse livers but much more abundant in humans. However, the clinical relevance of neutrophil populations in different TME subtypes in liver cancer remains unclear. Xue et al^6^ performed comprehensive single-cell-RNA sequencing analysis of 189 samples collected from 124 patients and 8 mice with liver tumors to understand different tumor immune microenvironment subtypes and validated their findings with multiplexed antibody staining. This study nicely presented various neutrophil populations spatially arranged with chemokine and cytokine networks in tumor control or tumor progression. Notably, tumor-associated neutrophils are significantly increased within the myeloid-cell–enriched subpopulation and PD-L1+ tumor-associated neutrophils suppressed T cell cytotoxicity, leading to unfavorable outcomes. In vivo studies showed that anti-Gr1 treatment to deplete neutrophils significantly reduced tumor progression. These findings also suggested that the diversity of immune subtypes in TME could be a difficulty to overcome to improve current immunotherapy.^7^

Moreover, immune-mediated adverse events have been considered as a barrier to the clinical benefit ICBs even in ICB-responding patients with HCC due to off-target issues and toxicity.^8^ In recent years, diverse efforts have been devoted to overcoming the off-target and nonresponder issues of cytokines through nanomedicine. This year, a few notable papers leveraged engineered cytokine therapies in liver-specific immune cells to eradicate liver cancer.^9^,^10^ IFNα is one candidate to target in HBV-associated liver cancer since it is required to induce various adaptive immune cells, including cytotoxic T cells in tumor control; however, pegylated IFN results in substantial immunotoxicity. One study developed a lentiviral vector platform containing a putative promoter sequence obtained from the mouse mannose receptor C-type 1 (*Mrc1*) gene to target liver-resident macrophages, termed KCs. Preclinical models showed rapid, sustained, and well-tolerated IFNα production and levels of toxicity, which activated antigen-presenting cells and CD8+ effector functions. On the other hand, it could increase IL-10 and CTLA-4–expressing CD4+ cells in orthotopic models. These exciting results suggest that the combination of anti-CTLA-4 with IFNα could synergize to produce a potent therapeutic response.

These highlighted new findings delineate the possibility for new targets of a-PD-L1/a-PD-1 treatment or new combination therapy. We anticipate that preclinical studies could help to improve ICB regimens while reducing side effects and lead to more effective patient stratification for ICB treatments. We hope that these novel findings will direct new avenues of immunotherapy in liver cancer to improve the survival rates of patients with HCC.

## References

[1] Villanueva A . Hepatocellular carcinoma. N Engl J Med. 2019;380:1450–62. 30970190 10.1056/NEJMra1713263

[2] Yang JD Hainaut P Gores GJ Amadou A Plymoth A Roberts LR . A global view of hepatocellular carcinoma: Trends, risk, prevention and management. Nat Rev Gastroenterol Hepatol. 2019;16:589–604. 31439937 10.1038/s41575-019-0186-y PMC6813818

[3] Sun LY Zhang KJ Xie YM Liu JW Xiao ZQ . Immunotherapies for advanced hepatocellular carcinoma. Front Pharmacol. 2023;14:1138493. 37025485 10.3389/fphar.2023.1138493 PMC10070708

[4] Moses L Pachter L . Museum of spatial transcriptomics. Nat Methods. 2022;19:534–546. 35273392 10.1038/s41592-022-01409-2

[5] Ruf B Bruhns M Babaei S Kedei N Ma L Revsine M . Tumor-associated macrophages trigger MAIT cell dysfunction at the HCC invasive margin. Cell. 2023;186:3686–705 e3632. 37595566 10.1016/j.cell.2023.07.026 PMC10461130

[6] Xue R Zhang Q Cao Q Kong R Xiang X Liu H . Liver tumour immune microenvironment subtypes and neutrophil heterogeneity. Nature. 2022;612:141–7. 36352227 10.1038/s41586-022-05400-x

[7] Spitzer MH Carmi Y Reticker-Flynn NE Kwek SS Madhireddy D Martins MM . Systemic immunity is required for effective cancer immunotherapy. Cell. 2017;168:487–502 e415. 28111070 10.1016/j.cell.2016.12.022 PMC5312823

[8] Ramadori P Gallage S Heikenwälder MF . Unique tumour microenvironment: when ferroptosis activation boosts ICI of liver cancer. Gut. 2023;72:1639–41. 37321831 10.1136/gutjnl-2023-329472

[9] Wang K Zhang X Ye H Wang X Fan Z Lu Q . Biomimetic nanovaccine-mediated multivalent IL-15 self-transpresentation (MIST) for potent and safe cancer immunotherapy. Nat Commun. 2023;14:6748. 37875481 10.1038/s41467-023-42155-z PMC10598200

[10] Kerzel T Giacca G Beretta S Bresesti C Notaro M Scotti GM . In vivo macrophage engineering reshapes the tumor microenvironment leading to eradication of liver metastases. Cancer Cell. 2023;41:1892–910 e1810. 37863068 10.1016/j.ccell.2023.09.014

